# Exploring Anesthesia Provider Preferences for Precision Feedback: Preference Elicitation Study

**DOI:** 10.2196/54071

**Published:** 2024-06-11

**Authors:** Zach Landis-Lewis, Chris A Andrews, Colin A Gross, Charles P Friedman, Nirav J Shah

**Affiliations:** 1 Department of Learning Health Sciences, University of Michigan, Ann Arbor, MI, United States; 2 Department of Ophthalmology and Visual Sciences, University of Michigan, Ann Arbor, MI, United States; 3 Biostatistics Department, University of Michigan, Ann Arbor, MI, United States; 4 Department of Anesthesiology, University of Michigan, Ann Arbor, MI, United States

**Keywords:** audit and feedback, dashboard, motivation, visualization, anesthesia care, anesthesia, feedback, engagement, effectiveness, precision feedback, experimental design, design, clinical practice, motivational, performance, performance data

## Abstract

**Background:**

Health care professionals must learn continuously as a core part of their work. As the rate of knowledge production in biomedicine increases, better support for health care professionals’ continuous learning is needed. In health systems, feedback is pervasive and is widely considered to be essential for learning that drives improvement. Clinical quality dashboards are one widely deployed approach to delivering feedback, but engagement with these systems is commonly low, reflecting a limited understanding of how to improve the effectiveness of feedback about health care. When coaches and facilitators deliver feedback for improving performance, they aim to be responsive to the recipient’s motivations, information needs, and preferences. However, such functionality is largely missing from dashboards and feedback reports. Precision feedback is the delivery of high-value, motivating performance information that is prioritized based on its motivational potential for a specific recipient, including their needs and preferences. Anesthesia care offers a clinical domain with high-quality performance data and an abundance of evidence-based quality metrics.

**Objective:**

The objective of this study is to explore anesthesia provider preferences for precision feedback.

**Methods:**

We developed a test set of precision feedback messages with balanced characteristics across 4 performance scenarios. We created an experimental design to expose participants to contrasting message versions. We recruited anesthesia providers and elicited their preferences through analysis of the content of preferred messages. Participants additionally rated their perceived benefit of preferred messages to clinical practice on a 5-point Likert scale.

**Results:**

We elicited preferences and feedback message benefit ratings from 35 participants. Preferences were diverse across participants but largely consistent within participants. Participants’ preferences were consistent for message temporality (α=.85) and display format (α=.80). Ratings of participants’ perceived benefit to clinical practice of preferred messages were high (mean rating 4.27, SD 0.77).

**Conclusions:**

Health care professionals exhibited diverse yet internally consistent preferences for precision feedback across a set of performance scenarios, while also giving messages high ratings of perceived benefit. A “one-size-fits-most approach” to performance feedback delivery would not appear to satisfy these preferences. Precision feedback systems may hold potential to improve support for health care professionals’ continuous learning by accommodating feedback preferences.

## Introduction

Health care professionals must learn continuously as a core part of their work. As the rate of knowledge production in biomedicine increases, better support for continuous learning is needed [1]. Feedback about care quality and outcomes is pervasive in health systems and widely considered to be essential for learning that drives improvement. Clinical performance feedback is one form of feedback that is commonly delivered to health care professionals in clinical quality dashboards and reports. However, engagement with these resources is generally low, and their impact has been less than optimal [2-5], resulting in missed opportunities to improve the quality and safety of care. A large proportion of randomized controlled trials of feedback interventions (also known as *audit and feedback*) show limited influence on clinical practice [5]. Moreover, what is considered as best practice for feedback interventions has not changed meaningfully for decades, even after hundreds of trials and repeated calls for new approaches to feedback interventions [6-8].

To our knowledge, most clinical performance feedback interventions use a “one-size-fits-most” approach to both the prioritization of performance information and its visual display as feedback, with the same metrics and visualizations being sent to all recipients. One-size-fits-most feedback may not be effective due to a host of characteristics such as individuals’ knowledge, skills, and motivational orientation to their work [2,3,9-11]. Methods used by coaches, educators, and quality improvement facilitators to deliver feedback suggest that these factors are important [2,12,13]. Furthermore, in the context of routine feedback interventions (eg, with monthly or quarterly measurement cycles), the value of performance information [14-16] may be reduced when performance is stable, but feedback interventions are not commonly prioritized accordingly. Given the increasing use and digitization of performance measures and clinical quality dashboards [17,18], health care systems need to understand how to better accommodate health care professionals’ feedback preferences and the corresponding value of performance information.

*Precision feedback* is feedback that has been prioritized based on its motivational potential for a specific recipient [19-23]. Using this approach, high-value feedback messages can be selected to enhance reports and emails, such as “You reached the top performer benchmark” and “Your performance dropped below the peer average.” The potential impact of precision feedback increases with greater variability and differences in individuals’ knowledge, skills, and motivational orientation, but these differences and their interactions are not well understood, as studies of health care professionals’ feedback preferences appear to be scarce. Qualitative studies have explored feedback preferences by asking participants to discuss their experiences with prior feedback; for example, they can be prompted by a published feedback report [24] or a performance report belonging to the participant or their organization [25]. Quantitative preference elicitation methods have been used extensively in health decision-making [26,27], but uncertainty about the measurement properties of preferences contributes to controversy around their use [28]. To our knowledge, no instruments of health care professional feedback preferences with validity evidence have been developed. To begin to explore and understand these differences, we designed a preference elicitation study for motivating performance information and its display format.

We conducted this study in the context of anesthesia care quality improvement. In this context, data generated about care processes are produced primarily by anesthesia machines that report the administration of anesthetics and the patient’s corresponding state with relatively high accuracy and reliability. Attribution of performance to individual anesthesia providers is feasible due to their authenticated use of an anesthesia machine for each operative case. A national-scale quality improvement consortium, the Multicenter Perioperative Outcomes Group (MPOG) [29,30], has developed approximately 70 performance measures for anesthesia care quality and outcomes. Feedback is delivered through its infrastructure via monthly emails and a clinical quality dashboard to more than 8000 health care professionals in more than 20 US states. Thus, a relatively large set of measures are routinely assessed using high-quality clinical data, representing performance information that health care professionals have limited natural sources for across their patient populations.

Multiple types of motivation are recognized as mechanisms through which feedback influences performance [2,10,11,31-33]. These various types of motivation can be understood as a consequence of the cognitive processing of performance information. We use the term *motivating performance information* to mean performance information that has the potential to motivate a feedback recipient through a known mechanism of action (Table 1). A key type of motivating performance information is a *comparison* that represents a discrepancy between the performance level of a feedback recipient and some *comparator* [22]. There are multiple types of comparators, including *benchmarks* having a performance level that is determined by a population-based analysis. Benchmarks are commonly calculated as a summary statistic of top performers, such as choosing the performance level for a population that occurs at the 90th percentile, or the achievable benchmark of care (ABC) method [34]. Another type of comparator is an *explicit target*, including goals or standards that set expectations for attaining a specific performance level that is not necessarily dependent upon peers or another reference group’s performance [35]. The choice of comparators can result in the use of alternate mechanisms of motivation, such as motivation related to social norms versus personal goal-setting. Another key type of motivating information is *trends* that represent change in performance (getting better or worse) [22]. Comparisons and trends may co-occur in performance data to represent an *achievement*, such as reaching a goal, or a *loss*, such as losing top-performer status [22].

**Table 1.** Glossary.

Term	Description	Source
Performance information	Information about measures, levels, time intervals, comparators, and feedback recipient	[20,22]
Feedback	Information about performance that can guide future action	[36]
Feedback recipient	A person, team, or organization to whom a feedback intervention is directed	[22]
Precision feedback	Feedback that is prioritized according to its motivational potential for a specific recipient	[23]
Motivating performance information	Performance information that holds motivational potential	[23]
Comparison	Motivating performance information that is about a discrepancy between the performance levels of a feedback recipient and a comparator	[22]
Trend	Motivating performance information that is about a change in performance	[22]
Achievement	Motivating performance information that is about a change from a negative comparison to a positive comparison	[22]
Loss	Motivating performance information that is about a change from a positive comparison to a negative comparison	[22]
Comparator	Information that is used to identify a discrepancy with the performance level of a feedback recipient	[22]
Benchmark	A comparator with a performance level that is calculated from the performance of other health professionals or peers	[22,35]
Explicit target	A comparator with a performance level that is explicitly expected	[22,35]
Time point information	Performance information that is about a single time interval	—^a^
Time series information	Performance information that is about multiple time intervals	—^a^
Causal pathway model	A specification of influential elements in a causal process, including preconditions, mechanisms, moderators, and outcomes	[37]

a Not available.

Comparisons and trends are represented using a wide range of visualizations in clinical quality dashboards and feedback reports [20]. These visualizations vary both in their content, such as the use of measures, comparators, and duration of time intervals, as well as the display format, such as bar charts, line charts, and tables to represent performance data. A review of published displays from feedback reports and dashboards identified 6 unique combinations of visualized performance information content [20]. For example, feedback displays vary in the number of performance measures, time intervals, and comparators that they visualize.

The display of feedback is theorized as one of many factors affecting the success of clinical performance feedback in Clinical Performance Feedback Intervention Theory (CP-FIT) [38], a leading theory of audit and feedback. Motivating performance information in clinical performance data concerns configurations of types of feedback display, but is also closely related to CP-FIT’s *goal* construct, which concerns the importance and relevance of feedback to health care professionals. Precision feedback may contribute to additional CP-FIT constructs, including health professional characteristics (knowledge and skills in quality improvement), feedback delivery (function), and implementation process (adaptability and ownership).

To understand anesthesia provider preferences for motivating performance information and feedback display format, we investigated the following four research questions:

1. To what extent do anesthesia providers’ selected messages reveal an overall preference for
  - messages containing time series versus time point information (temporality)?
  - messages relative to benchmarks versus explicit performance targets (basis of comparison)?
  - messages formatted as bar charts versus line charts and text only (display format)?
2. How consistent are individual anesthesia provider preferences?
3. To what extent do anesthesia provider preferences depend on performance level, trend, and their professional background?
4. To what extent are preferred feedback messages perceived to hold potential to improve future clinical practice?

## Methods

### Overview

To address these questions, we developed a test set of feedback messages that a software application could generate. We formatted these as brief email messages, but designed them as “least common denominator” content that could also be delivered via other channels for feedback, such as clinical quality dashboards.

In the absence of instruments with validity evidence for assessing health care professional feedback preferences, we created an experimental design to elicit preferences that would expose participants, who were anesthesia providers, to contrasting message versions. To enable measurement validity assessment, we developed performance scenarios in which the same motivating performance information and display characteristics could be repeated in contrasting messages.

### Ethical Considerations

This study was approved by the University of Michigan Health Sciences and Behavioral Sciences Institutional Review Board (IRB-HSBS HUM00167426). All participants provided consent to participate and were informed about the ability to opt out of the study. No participant identifiers were collected with the research data for this study, preventing the linking of participants’ responses with their identities. No incentives for participation were provided. We offered participants an opportunity to receive a copy of the study results upon completion.

### Email Test Set Development

**Figure 1.**
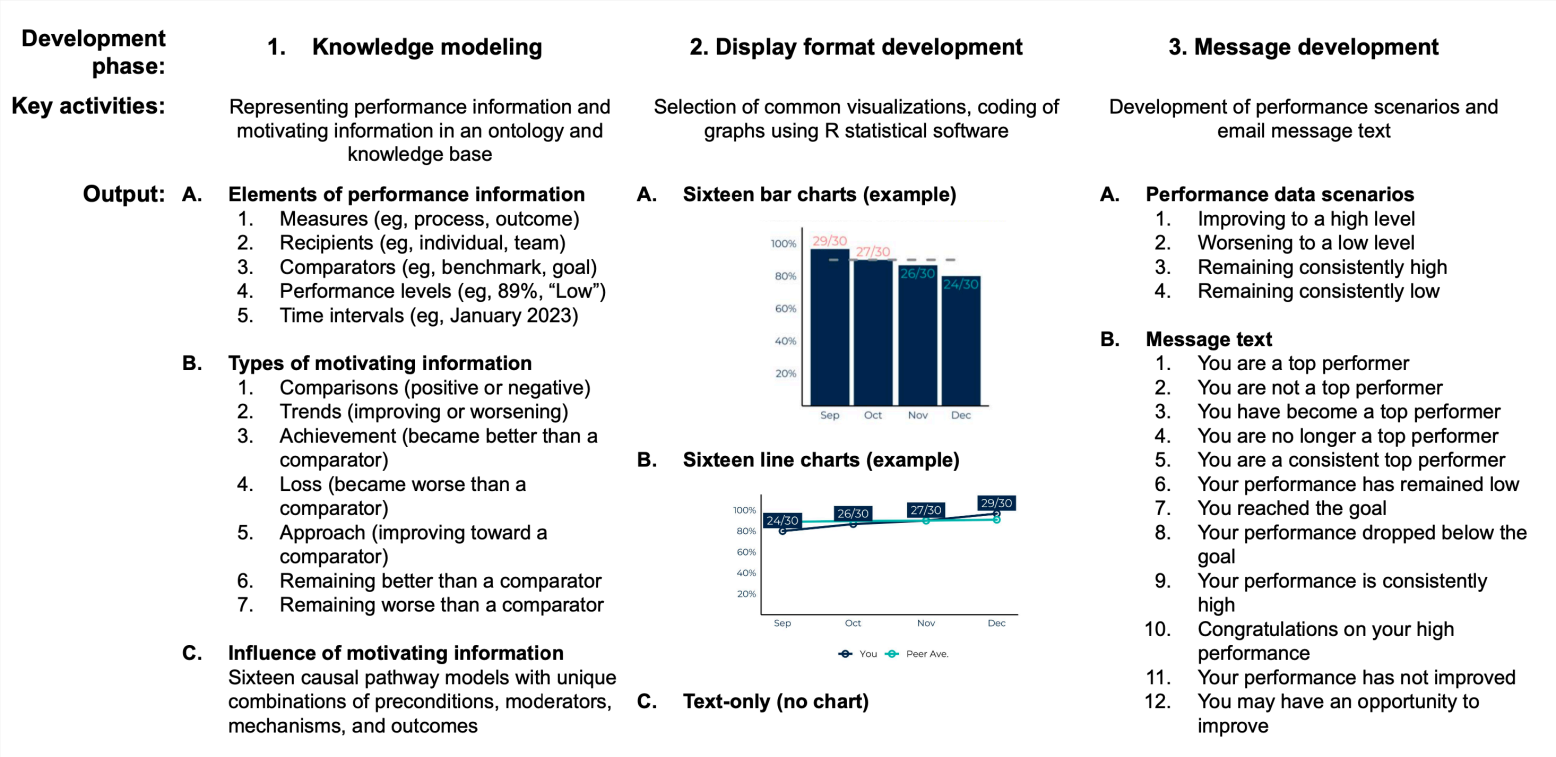
Development of a precision feedback email test set.

We developed the email message test set iteratively in three phases: (1) knowledge modeling, (2) display format development, and (3) message set development (Figure 1).

#### Phase 1: Knowledge Modeling

In the first phase we modeled knowledge about the elements of performance information, types of motivating information, and the influence of motivating performance information (Figure 1). We iteratively refined a model of the elements of performance information through an analysis of published feedback reports [20], resulting in the identification of 5 key elements: measures, recipients, comparators, performance levels, and time intervals. We developed a model of motivating information that combined the 5 elements of performance information into types of motivating information, including comparisons, trends, achievement, and loss. Each type of motivating information was defined using the elements of performance information. For example, a comparison (a kind of motivating performance information) was defined as a discrepancy between the performance levels of a feedback recipient and a comparator.

Through modeling types of motivating performance information, we recognized that the choice of comparator could affect which type of motivation was used to influence a recipient. For example, choosing a 90th-percentile peer benchmark as a comparator does not necessarily leverage motivation from goal-setting when recipients do not form an intention to reach the benchmark as their personal goal. By inviting anesthesia providers to set goals, feedback that shows performance improving toward a goal may leverage motivation arising from a desire for growth and achievement, rather than a desire for safety and avoidance of harm. These sources of motivation can differentially interact with the feedback sign (ie, valence) to have counterintuitive effects, such as goal abandonment, relaxation, or the delivery of low-value feedback [2,10].

To understand how different types of motivating performance information might relate to theoretical mechanisms of influence, we created causal pathway models [37] for each type of motivating information with benchmark and explicit target comparators (Multimedia Appendix 1). For example, in one causal pathway we modeled the expected influence of a feedback intervention that combines three elements of a recipient’s performance: (1) performance below a comparator (low performance level), (2) a benchmark (such as a peer average), and (3) performance getting better (improving trend). This pathway could represent the influence of precision feedback emails that show performance approaching a peer average, which could indicate to recipients that efforts to improve performance appear to be succeeding. Based on the theoretical construct of *positive velocity* [31] (ie, showing performance improvement), this causal pathway (which we named *social approach* due to the recipient reducing a performance gap with a peer benchmark) uses motivation as a mechanism of action, through which a feedback recipient may decide to increase or sustain effort to improve performance.

We drafted and refined example messages for each type of motivating information. For the causal pathway *social approach*, an example message is “Your performance is approaching the benchmark.” We implemented the causal pathway models in computer-interpretable form in a knowledge base to enable automation of the processing of performance information to identify motivating information in a precision feedback system.

#### Phase 2: Display Format Development

In the second phase we developed display formats for motivating information in the body of an email message. We selected visualizations (ie, bar charts and line charts) common in health care organizations so that a familiar format would convey the minimal amount of information necessary for each causal pathway. We developed software to generate visualizations within an email message using R (version 4.3.3; R Foundation for Statistical Computing). We included the absence of a visualization (ie, text only) to accommodate recipient preferences for concise, text-based communication (Figure 1).

#### Phase 3: Message Set Development

In the third and final phase we created a test set of email messages with balanced characteristics of motivating information and display formats. We began by creating four performance scenarios with alternate performance levels (high vs low) and trends (improvement vs worsening vs stable): (1) improvement to a high level, (2) worsening to a low level, (3) consistently high (stable) performance, and (4) consistently low (stable) performance (Table 2). In all scenarios, the recipient’s performance could be compared with either the peer average (benchmark comparator) or an organizational goal (explicit target comparator). We set the recipient’s performance level to have the same relationship with each comparator (better or worse), enabling either comparator to be displayed while maintaining balance with other elements.

**Table 2.** Precision feedback email message test set specification.

Performance data scenario			Motivating information characteristics		Key message	Display format	
Level	Trend	Performance description	Temporality	Comparator		Shown to group A	Shown to group B
High	Improving	Performance level moves above comparators	Time series	Benchmark	You have become a top performer	Line chart	Bar chart
				Explicit target	You reached the goal	Bar chart	Line chart
			Time point	Benchmark	You are a top performer	Bar chart	Text only
				Explicit target	Congratulations on your high performance	Text only	Bar chart
Low	Worsening	Performance level moves below comparators	Time series	Benchmark	You are no longer a top performer	Bar chart	Line chart
				Explicit target	Your performance dropped below the goal	Line chart	Bar chart
			Time point	Benchmark	You are not a top performer	Text only	Bar chart
				Explicit target	You may have an opportunity to improve	Bar chart	Text only
High	No change	Performance level is consistently above comparators	Time series	Benchmark	You are a consistent top performer	Bar chart	Line chart
				Explicit target	Your performance is consistently high	Line chart	Bar chart
			Time point	Benchmark	You are a top performer	Text only	Bar chart
				Explicit target	Congratulations on your high performance	Bar chart	Text only
Low	No change	Performance level is consistently below comparators	Time series	Benchmark	Your performance has remained low	Line chart	Bar chart
				Explicit target	Your performance has not improved	Bar chart	Line chart
			Time point	Benchmark	You are not a top performer	Bar chart	Text only
				Explicit target	You may have an opportunity to improve	Text only	Bar chart

We selected types of motivating information and their example messages across three characteristics: (1) performance temporality (time series vs time point), (2) performance comparison basis (benchmark vs explicit target), and (3) performance display format (bar chart or other). We selected the bar chart format as a key display format because of its common use in health care organizations. We further divided the *other* display format into *line chart* and *text only*. We composed emails with example messages from each type, based on a single quality measure (Avoiding postoperative nausea and vomiting [PONV-03]) for anesthesia providers. The resulting emails contained information from the same performance scenarios, but not all information from each scenario was provided in each message. For example, of the 4 emails that each participant read in each scenario, 2 messages contained a goal comparator (explicit target), while the other 2 messages showed a peer benchmark comparator instead.

### Study Design

We designed a within-subjects, repeated measures study of anesthesia provider preferences for precision feedback using a test set of prototype email messages printed on paper. We created 2 versions of the test set with alternate display formats for each message (group A vs group B) to enable randomization of the pairing of display format with motivating information. We created a document containing all of the email messages in the test set (Multimedia Appendix 2). We printed paper copies of the messages and organized them into packets in varying order for a paper card selection task. Based on our experience, we estimated that a sample of more than 30 participants would provide adequate power to detect meaningful differences in summary statistics and internal consistency of preferences.

### Population and Setting

We recruited anesthesia providers from a single academic medical center in the midwestern United States. Anesthesiologists (physicians) and certified registered nurse anesthetists (CRNAs) were eligible to participate. A member of the study team recruited anesthesia provider participants by email. All participants received monthly anesthesia provider feedback emails from MPOG.

### Data Collection

Upon enrollment, we scheduled a 15-minute proctored video call with each participant and sent them a paper packet with email prototypes before the call. Participants were randomized to receive a paper packet of messages from either group A or group B of the message test set, each of which contained 16 email messages grouped in 4 packets of 4. Each packet of 4 messages contained alternate message formats for 1 of 4 performance scenarios, with balanced message formats and performance information across the 4 scenarios. We created a questionnaire to collect data from participants about their preferred emails using Qualtrics (Qualtrics, Inc). We created 2 versions of the questionnaire (A and B), 1 for each message group to be used based on the participant’s random assignment at the time of enrollment. At the time of enrollment, we also instructed participants to have a desk space or table available for placing printed email messages in front of them, and to wait to open the packets until asked to do so during the video call.

### Preference Elicitation and Message Usability Assessment

At the start of the proctored video call, a research team member introduced the study, confirmed the participant’s preparation, and provided a link to the questionnaire. During the completion of the questionnaire, the participant repeated a preferred email message selection task 4 times, following the instructions in their packet, once for each performance data scenario. The questionnaire software randomized the scenario presentation order. We described the scenarios as hypothetical performances that the participant could imagine as being their own. At the start of each scenario, participants were asked to find the corresponding set of emails, identified with a cover sheet. Participants were then asked to lay out all 4 of the printed email messages for that scenario in front of them. Next, participants read each message and selected their preferred message. After selecting a preferred message, participants responded to the following statement: “I gained information from this email that would benefit my practice.” We adapted this question from an instrument with good validity evidence for assessing the usability of feedback displays [39]. Responses were collected on a 5-point Likert scale, ranging from strongly disagree to strongly agree. The survey questions did not ask directly about preference for information content or display format. Instead, participants’ preferences were inferred through the types of content and display format that the selected message contained. After participants completed the questionnaire, we conducted brief interviews and collected qualitative data that were analyzed separately and will be reported elsewhere.

### Analysis

To identify preferences, we analyzed 2 characteristics of the selected messages: motivating information (including temporality type and comparator type) and display format. We summed the selected messages with each type of motivating information and display format and calculated descriptive statistics for these sums (Q1). To investigate the consistency of participants’ preferences, we calculated the Cronbach α for each preference characteristic in participants’ selected messages across the 4 performance scenarios (Q2). We used descriptive statistics to assess relationships between participants’ preferences and the characteristics of the 4 performance scenarios, including performance level (high vs low) and trend presence (present vs absent). Similarly, we considered relationships between participants’ preferences and their professional background using descriptive statistics (Q3).

To understand participants’ perceptions of the potential benefit of precision feedback to their clinical practice, we analyzed ratings of perceived benefit for selected messages using descriptive statistics (Q4). We conducted analyses using R and Google Sheets (Google LLC).

## Results

We recruited 35 anesthesia providers, including 18 anesthesiologists and 17 CRNAs (Table 3). All participants completed all message selection tasks, resulting in the selection of 140 preferred precision feedback messages.

**Table 3.** Study participant characteristics (N=35).

Characteristics		Participants, n (%)
**Professional role**		
	Anesthesiologist	18 (51)
	Certified registered nurse anesthetist	17 (49)
**Race/ethnicity**		
	African-American	0 (0)
	Asian	2 (6)
	Hispanic	0 (0)
	White	31 (89)
	Other	2 (6)
**Gender**		
	Female	19 (54)
	Male	16 (46)
	Nonbinary/other	0 (0)

### To What Extent Do Anesthesia Providers’ Selected Messages Reveal an Overall Preference for Temporality (Q1a), Basis of Comparison (Q1b), and Display Format (Q1c)?

An overall preference for multiple time intervals (ie, time series) was apparent, with 110 of 140 (79%) messages being selected over those with a single time interval (ie, time point) (Q1a). Preferences for display format were highly varied, with selected messages being equally distributed between bar charts versus other formats (Table 4) (Q1c). Preferred messages were also highly varied in their comparators, with 74 of 140 (53%) preferred cards containing explicit target comparators (ie, organizational goals not dependent on population performance) (Q1b), but our assessment of the consistency suggests that the comparator result was not reliable as a preference characteristic (see Q2 below).

**Table 4.** Characteristics of preferred precision feedback messages.

Message characteristic and subtype		Preferred messages (n=140), n (%)	Message characteristic preference (n=4), mean (SD)	α
**Temporality**				.85
	Time series	110 (79)	3.14 (1.38)	
	Time point	30 (21)	0.86 (1.38)	
**Comparators**				–.40
	Benchmark	66 (47)	1.89 (0.87)	
	Explicit target	74 (53)	2.11 (0.87)	
**Display format**				.80
	Bar chart	70 (50)	2.00 (1.61)	
	Other display	70 (50)	2.00 (1.61)	

### How Consistent Are Individual Anesthesia Provider Preferences (Q2)?

Participants’ preferences were consistent for temporality (α=.85) and display format (α=.80). For performance comparators, participants’ selected messages were negatively correlated (α=−.40), indicating an absence of consistency, perhaps from an incorrect measurement model [40]. We consider this result to be an artifact of the study design, given that our message test set balanced several characteristics and created opportunities to select them in combination. We anticipate that comparators were not salient for participants, relative to the visual display and temporality characteristics; therefore, we are unable to draw conclusions about preferences for comparators.

### To What Extent Do Anesthesia Provider Preferences Depend on Performance Level and Trend and Their Professional Background (Q3)?

Participant preferences for temporality and display format did not appear to depend on the messages’ performance level, with relatively similar means for the selection of each type of message content. Similarly, these preferences did not appear to vary with the presence or absence of performance trends (Table 5).

**Table 5.** Precision feedback preferences by performance scenario characteristics.

	Temporality preference, mean (SD)		Comparator preference, mean (SD)		Display format preference, mean (SD)			
	Time series	Time point	Benchmark	Explicit target	Bar chart	Other display	Other display: line chart	Other display: text only
Level: high	1.60 (0.69)	0.40 (0.69)	1.09 (0.56)	0.91 (0.56)	0.94 (0.87)	1.06 (0.87)	0.77 (0.88)	0.29 (0.62)
Level: low	1.54 (0.78)	0.46 (0.78)	0.80 (0.53)	1.20 (0.53)	1.06 (0.87)	0.94 (0.87)	0.66 (0.84)	0.29 (0.62)
Trend present	1.66 (0.68)	0.34 (0.68	1.06 (0.54)	0.95 (0.54)	1.09 (0.89)	0.91 (0.89)	0.69 (0.83)	0.23 (0.60)
Trend absent	1.49 (0.78)	0.51 (0.78)	0.83 (0.51)	1.17 (0.51)	0.91 (0.85)	1.09 (0.85)	0.74 (0.85)	0.34 (0.64)

Preferences for temporality and display format varied with participants’ professional background (Table 6). Some professional role-based differences in means were apparent, such as a higher preference for time point messages among CRNAs than anesthesiologists (mean message characteristics preference 1.18, SD 1.59 vs mean message characteristic preference 0.56, SD 1.10). However, a majority of CRNAs preferred time series messages, and all message characteristics were repeatedly observed in selections by participants from both professional background–based groups.

**Table 6.** Precision feedback preferences by professional background.

	Temporality preference, mean (SD)		Comparator preference, mean (SD)		Display format preference, mean (SD)			
	Time series	Time point	Benchmark	Explicit target	Bar chart	Other display	Other display: line chart	Other display: text only
Anesthesiologist	3.44 (1.10)	0.56 (1.10)	1.83 (0.79)	2.17 (0.79)	2.28 (1.60)	1.72 (1.60)	1.33 (1.68)	0.39 (0.78)
Certified registered nurse anesthetist	2.82 (1.59)	1.18 (1.59)	1.94 (0.97)	2.06 (0.97)	1.71 (1.61)	2.29 (1.61)	1.53 (1.55)	0.76 (1.48)

### To What Extent Are Preferred Feedback Messages Perceived to Hold Potential to Improve Future Clinical Practice (Q4)?

Participants’ ratings of perceived benefit from all precision feedback messages were positive, with a mean rating of 4.27 (SD 0.77). Although positive overall, the anesthesiologists’ ratings were lower than the CRNAs’ ratings (mean rating 4.08, SD 0.85 vs mean rating 4.47, SD 0.61). Ratings for messages did not appear to vary across performance levels or with trends (Table 7). Average ratings of perceived benefit were similar across message content characteristics. One exception to this was for explicit target comparators, which appeared to receive slightly higher ratings (mean rating 4.38, SD 0.7) over benchmark comparators (mean rating 4.15, SD 0.83).

**Table 7.** Perceived benefit of selected messages.

Characteristics			Mean rating (SD)
**Participant professional background**			
	Anesthesiologist		4.08 (0.85)
	Certified registered nurse anesthetist		4.47 (0.61)
**Performance scenario**			
	**Performance level**		
		High performance	4.23 (0.76)
		Low performance	4.31 (0.77)
	**Performance trend**		
		Trend present	4.34 (0.72)
		Trend absent	4.2 (0.81)
**Message content**			
	**Temporality**		
		Time series	4.27 (0.81)
		Time point	4.27 (0.58)
	**Comparator**		
		Benchmark	4.15 (0.83)
		Explicit target	4.38 (0.7)
	**Display format**		
		Bar chart	4.27 (0.76)
		Other display	4.27 (0.78)
		Other display: Line chart	4.28 (0.83)
		Other display: text only	4.25 (0.64)

## Discussion

### Principal Results

In this study, we found that anesthesia provider preferences for motivating information and display format varied, which suggests that individual difference characteristics may represent a barrier to improving the effectiveness of feedback interventions. Across a set of 4 diverse performance scenarios, we observed preference variability that precision feedback could better address than one-size-fits-most feedback in this anesthesia provider population.

We observed consistency in participant preferences for the temporality of motivating information and for display format. Even though a large majority of participants preferred messages with time-series information, the participants who preferred time-point messages reliably selected them. The consistency of preferences for display format was similar, and also more varied, with exactly half of participants choosing bar charts over other visual displays. We also did not observe differences in preferences associated with performance scenario characteristics or professional background that could be used to design one-size-fits-most feedback interventions.

While participants exhibited diverse preferences, their ratings of the benefit of the messages were consistently high across performance scenarios. These findings suggest that anesthesia providers would welcome the enhancement of feedback interventions with precision feedback that prioritizes motivating information. These findings are important because they point to a possible approach for improving audit and feedback that can leverage both high and low performance, as well as increasing or decreasing trends, to prioritize performance feedback.

To our knowledge, this is the first quantitative study of preferences for clinical performance feedback. As an exploratory study, the findings primarily demonstrate the existence of differences in preferences for feedback, rather than speaking to the significance of their role in the success of clinical performance feedback. Our findings are related to CP-FIT, which recognizes that health professional knowledge and skills for engaging with feedback can be important factors for the success of feedback [38]. Differences in feedback preferences could be driven by differences in health care professionals’ knowledge and skills related to the interpretation of performance data. For example, participants’ variable and consistent selection of messages could be related to their graph literacy skills [41,42]. Precision feedback could be used to accommodate these and other individual differences by enabling health professionals to configure their feedback delivery and display, which further holds potential to increase feelings of ownership of feedback. By prioritizing motivating information according to recipients’ preferences, precision feedback could be a strategy for reducing the cognitive load required by health professionals to recognize and assess the priority of learning opportunities. Precision feedback has also potential to improve feedback cycle completion by delivering information that is more likely to be perceived and accepted, resulting in increased formation of intentions to sustain or improve performance. In terms of CP-FIT, precision feedback can be understood as an approach for prioritization of feedback messages that are more likely to result in successful completion of the feedback cycle.

Our findings are aligned with the idea that positive feedback can be effective for learning and improvement [13], as well as sustainment of high performance. It is noteworthy that participants rated precision feedback messages as beneficial even when performance was high, such as the messages “you are a top performer” or “you reached the goal.” This finding points to the possibility that a key function of feedback may be to motivate recipients through appreciation of accomplishments [43], including recognition of high performance, in addition to motivating recipients to learn to improve.

### Limitations

As an exploratory study for a novel type of feedback intervention, there are several important limitations for this study. The poor consistency of preferences demonstrated for performance comparators suggests that participants did not meaningfully differentiate between peer-based benchmarks and explicit targets, as presented in the message test set. This may be a function of the labels used for the comparators message test set, and during the study we discovered that some of the printed messages contained the abbreviation “ave” instead of “avg” for the peer average comparator. Competing explanations are that (1) anesthesia providers equated the value of both comparator types or did not perceive them as fundamentally different, and (2) that this characteristic was less salient than the others, such that its significance was negligible.

Using performance scenarios based on synthetic performance data may have introduced bias in participants’ responses. However, the consistency of participant preferences for temporality of motivating information and display format suggests that this bias was not significant. Nevertheless, our study design assessed preferences within types of motivating information (eg, high and improving performance or low and worsening performance) that were presented with unambiguous motivating information, such as trends showing marked improvement or worsening. As such, our results do not address the appropriateness of using performance scenarios to elicit the strength of anesthesia provider preferences directly; rather, they primarily demonstrate the existence of individual differences as an exploration of factors that may moderate the influence of feedback on health care professional learning and improvement.

We asked participants to rate the perceived benefit of messages that they had already selected as their preferred message, which may have resulted in positively biased ratings. Furthermore, we used a single performance measure for all messages (avoiding postoperative nausea and vomiting) that may not be representative of other performance measures, both in terms of perceived benefit and preferences for motivating information. We did not evaluate feedback about clinical outcome measures, which may have resulted in a different preference profile across this population. We also did not evaluate participants’ skills or knowledge to engage effectively in feedback, which is a recognized factor [38] that may have resulted in further insight into participant preferences.

Additional limitations include the context and nature of the preference elicitation task, which was done in a video call with paper prototypes and thus differed from the context of email use in health care organizations. When designing this study, we chose to use email messages printed on paper because we could not identify a remote, video call–proctored approach that would allow participants to consider 4 different messages types in the same field of view on their personal or work computer without a risk of technical complications from participants’ particular computer monitor and device configurations.

Our model of preferences in this study was linear and static and assumed that available information was complete, but anesthesia provider preferences may be nonlinear, dynamic, and depend on missing information that we did not consider. When designing the test set of messages, we paired the text-only display format consistently with time-point information, and line charts with the time-series format. As such, preferences for line charts and text-only display formats were not independent from temporality. We recruited anesthesia providers from a single academic institution whose population is not necessarily representative of other anesthesia provider populations. We did not recruit any anesthesia providers who identified as Black or Hispanic, increasing the likelihood that our results are racially and ethnically biased toward the perspectives of anesthesia providers who identify as White and non-Hispanic. In spite of all of these limitations, we note that the variability that we observed demonstrates that preferences were nonuniform in this small population, which suggests that a one-size-fits-all solution may be inadequate for feedback reporting to anesthesia providers more generally.

### Future Studies

We anticipate that preference clusters may exist and may be identifiable in studies that are better powered to detect such differences. Such clusters could be used to develop profiles for precision feedback, such as profiles for anesthesia providers who prefer text-only messages about low performance or those who prefer visualization of performance changes (ie, trends) using time-series displays in line charts. Future studies may be able to detect preference clusters to better understand the diversity of preferences for performance feedback across a larger anesthesia provider population that is more racially, ethnically, and geographically diverse. Furthermore, we would welcome studies that aim to better understand the diversity of anesthesia provider preferences in association with additional anesthesia provider characteristics, such as duration of professional experience, clinical setting, and organization type.

### Conclusions

Clinical performance feedback to health care professionals has potential to support continuous learning and influence practice, but this potential is frequently not achieved. By prioritizing motivating performance information based on the preferences and needs identified for a health care professional population, precision feedback may increase the effectiveness of clinical performance feedback for health care professionals’ continuous learning and resulting quality improvement. Among a sample of anesthesia providers, preferences for precision feedback were varied, yet consistent within participants. Furthermore, participants’ perceived benefits of precision feedback messages were observed to be high across a diverse set of performance scenarios. Based on these findings, it appears that precision feedback holds potential to improve support for health care professionals’ continuous learning.

## Supplementary material

10.2196/54071 Multimedia Appendix 1 Causal pathway models for precision feedback interventions.

10.2196/54071 Multimedia Appendix 2 Test set of precision feedback emails.

10.2196/54071 Multimedia Appendix 3 Study data.

10.2196/54071 Checklist 1 STROBE (Strengthening the Reporting of Observational Studies in Epidemiology) statement.

## Abbreviations

ABC: achievable benchmark of care
CP-FIT: Clinical Performance Feedback Intervention Theory
CRNA: certified registered nurse anesthetist
MPOG: Multicenter Perioperative Outcomes Group
