# Supplementary material for: Exploring Anesthesia Provider Preferences for Precision Feedback: Preference Elicitation Study
Source: JMIR Med Educ. 2024 Jun 11;10:e54071. doi: 10.2196/54071 (PMC11185285; doi:10.2196/54071)
Supplement: Multimedia Appendix 1 [file mededu-v10-e54071-s001.pdf]

Appendix 1: Causal pathways for precision feedback

#	Name	Feedback intervention description	Example precision feedback message	Information content			Preconditions	Moderators (cognitive)	Mechanisms of action*	Foundational theories	Proximal outcome
				Comparator type	Performance level	Trend					
1	Social better	Show performance that is better than a social comparator	You are a top performer	Social	High	None	Positive gap, social comparator	Habituation, regulatory fit, gap size	Knowledge, subjective norms, motivation	Regulatory Fit Theory	Performance improvement or sustainment
2	Social gain	Show performance becoming better than a social comparator	You reached the benchmark this month			Increasing	Positive gap, social comparator, positive trend, achievement	Habituation, regulatory fit, gap size, slope of trend, time since last achievement			
3	Social remain better	Show performance remaining better than a social comparator	Congratulations on your consistently high performance			None	Positive gap, consecutive positive gaps, social comparator	Habituation, regulatory fit, gap size, time since last achievement			
4	Social worse	Show performance that is worse than a social comparator	You are not a top performer		Low	None	Negative gap, social comparator	Habituation, regulatory fit, gap size		Control Theory, Regulatory Fit Theory, Feedback Intervention Theory	
5	Social loss	Show performance becoming worse than a social comparator	You are no longer a top performer			Decreasing	Negative gap, social comparator, negative trend, loss	Habituation, regulatory fit, gap size, slope of trend, time since last loss			
6	Social remain worse	Show performance remaining worse than a social comparator	Your performance has remained low			None	Negative gap, consecutive negative gaps, social comparator, negative trend	Habituation, regulatory fit, gap size			
7	Social approach	Show performance approaching a social comparator	Your performance is approaching the benchmark			Increasing	Negative gap, social comparator, positive trend	Habituation, regulatory fit, gap size, slope of trend	Knowledge, subjective norms, optimism, motivation		
8	Goal better	Show performance that is better than a goal	Your performance is above the goal	Goal	High	None	Positive gap, goal comparator	Habituation, regulatory fit, gap size	Knowledge, goals, motivation	Regulatory Fit Theory	
9	Goal gain	Show performance becoming better than a goal	You reached the goal this month			Increasing	Positive gap, goal comparator, positive trend, achievement	Habituation, regulatory fit, gap size, slope of trend, time since last achievement			
10	Goal remain better	Show performance remaining better than a goal	Your performance is consistently above the goal			None	Consecutive positive gaps, goal comparator	Habituation, regulatory fit, gap size, time since last achievement			
11	Goal worse	Show performance that is worse than a goal	You may have an opportunity to improve		Low	None	Negative gap, goal comparator	Habituation, regulatory fit, gap size		Goal-Setting Theory, Regulatory Fit Theory, Feedback Intervention Theory	
12	Goal loss	Show performance becoming worse than a goal	Your performance dropped below the goal			Decreasing	Negative gap, goal comparator, negative trend, loss	Habituation, regulatory fit, gap size, slope of trend, time since last loss			
13	Goal remain worse	Show performance remaining worse than a goal	You have not yet reached the goal			None	Negative gap, consecutive negative gaps, goal comparator, negative trend	Habituation, regulatory fit, gap size			
14	Goal approach	Show performance approaching a goal	Your performance is approaching the goal			Increasing	Negative gap, goal comparator, positive trend	Habituation, regulatory fit, gap size, slope of trend	Knowledge, goals, optimism, motivation		
15	Improving	Show performance improving	Your performance is improving	None	Unknown	Increasing	Positive trend	Habituation, regulatory fit, gap size	Knowledge, optimism, motivation	Regulatory Fit Theory, Feedback Intervention Theory	
16	Worsening	Show performance worsening	Your performance is getting worse			Decreasing	Negative trend		Knowledge, motivation		

*Mechanisms are based on Carey et al 2019 (https://doi.org/10.1093/abm/kay078).

From these mechanisms, "Feedback processes" was excluded because its definition (Processes through which current behavior is compared against a particular standard) would establish circular logic in all relevant conditions.
