# Supplementary material for: Exploring Anesthesia Provider Preferences for Precision Feedback: Preference Elicitation Study
Source: JMIR Med Educ. 2024 Jun 11;10:e54071. doi: 10.2196/54071 (PMC11185285; doi:10.2196/54071)
Supplement: Multimedia Appendix 2 [file mededu-v10-e54071-s002.pdf]

Card 1A

Dear Alex,

You have become a top performer this month for the measure postoperative nausea and vomiting (PONV 03):

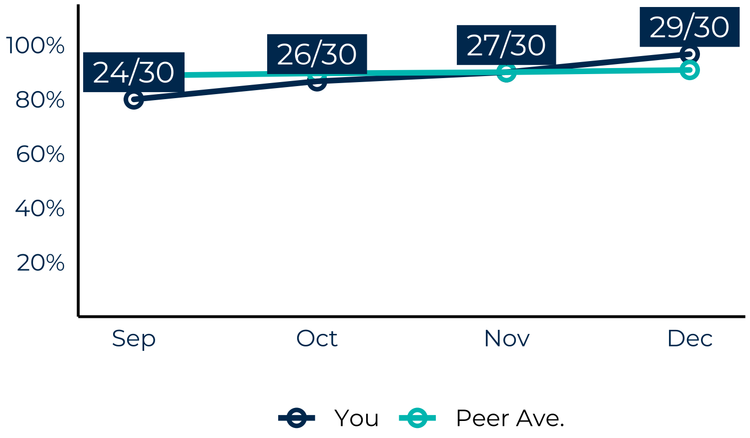

A case-by-case breakdown of your results are available at your clinical quality dashboard.

More information about the rationale for the measure PONV 03 and how it is calculated are available here.

Sincerely,

The MPOG Quality Team

…

Card 1B

Dear Alex,

You have become a top performer this month for the measure postoperative nausea and vomiting (PONV 03):

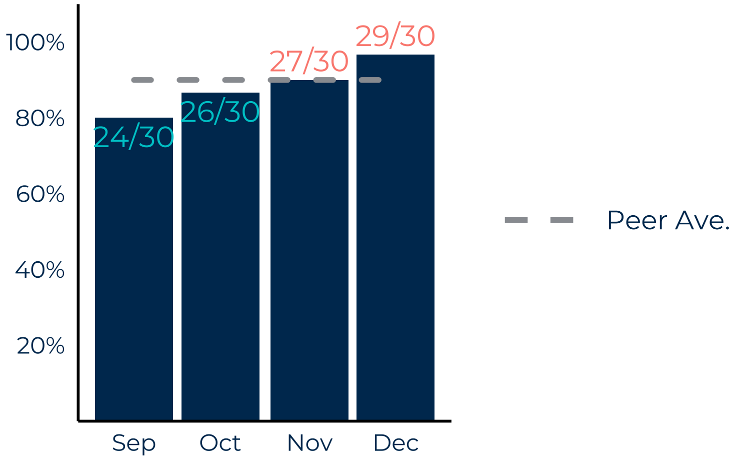

A case-by-case breakdown of your results are available at your clinical quality dashboard.

More information about the rationale for the measure PONV 03 and how it is calculated are available here.

Sincerely,

The MPOG Quality Team

Card 2A

Dear Alex,

You reached the goal this month for the measure postoperative nausea and vomiting (PONV 03):

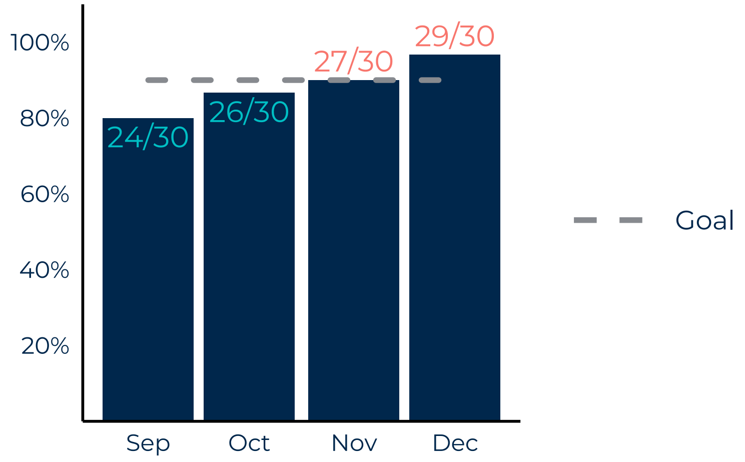

A case-by-case breakdown of your results are available at your clinical quality dashboard.

More information about the rationale for the measure PONV 03 and how it is calculated are available here.

Sincerely,

The MPOG Quality Team

…

Card 2B

Dear Alex,

You reached the goal this month for the measure postoperative nausea and vomiting (PONV 03):

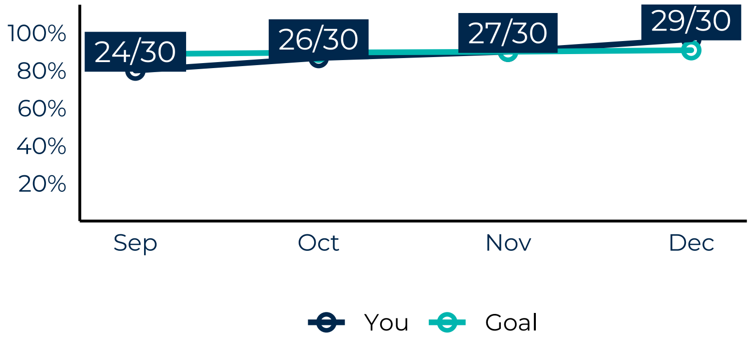

A case-by-case breakdown of your results are available at your clinical quality dashboard.

More information about the rationale for the measure PONV 03 and how it is calculated are available here.

Sincerely,

The MPOG Quality Team

Card 3A

Dear Alex,

You are a top performer this month for the measure postoperative nausea and vomiting (PONV 03):

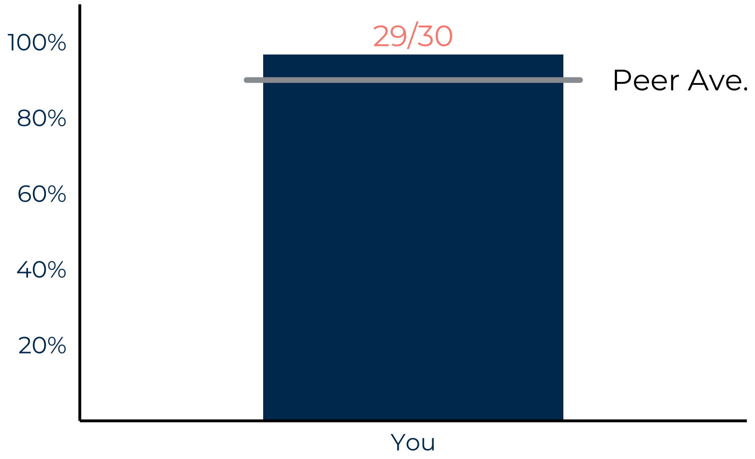

A case-by-case breakdown of your results are available at your clinical quality dashboard.

More information about the rationale for the measure PONV 03 and how it is calculated are available here.

Sincerely,

The MPOG Quality Team

…

Card 3B

Dear Alex,

You are a top performer this month for the measure postoperative nausea and vomiting (PONV 03).

Your performance was 96.7% (29/30), above the peer average of 90% (27/30).

A case-by-case breakdown of your results are available at your clinical quality dashboard.

More information about the rationale for the measure PONV 03 and how it is calculated are available here.

Sincerely,

The MPOG Quality Team

Card 4A

Dear Alex,

Congratulations on your high performance this month for the measure postoperative nausea and vomiting (PONV 03).

Your performance was 96.7% (29/30), above the goal of 90% (27/30).

A case-by-case breakdown of your results are available at your clinical quality dashboard.

More information about the rationale for the measure PONV 03 and how it is calculated are available here.

Sincerely,

The MPOG Quality Team

…

Card 4B

Dear Alex,

Congratulations on your high performance this month, 29/30, for the measure, postoperative nausea and vomiting (PONV 03):

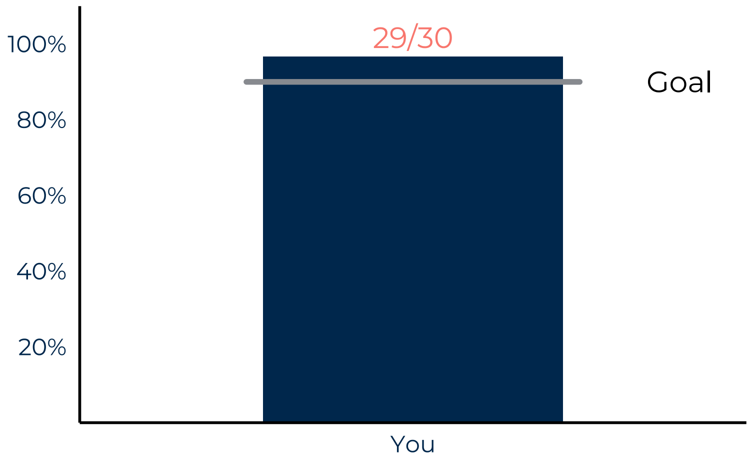

A case-by-case breakdown of your results are available at your clinical quality dashboard.

More information about the rationale for the measure PONV 03 and how it is calculated are available here.

Sincerely,

The MPOG Quality Team

Card 5A

Dear Alex,

You are no longer a top performer for the measure postoperative nausea and vomiting (PONV 03):

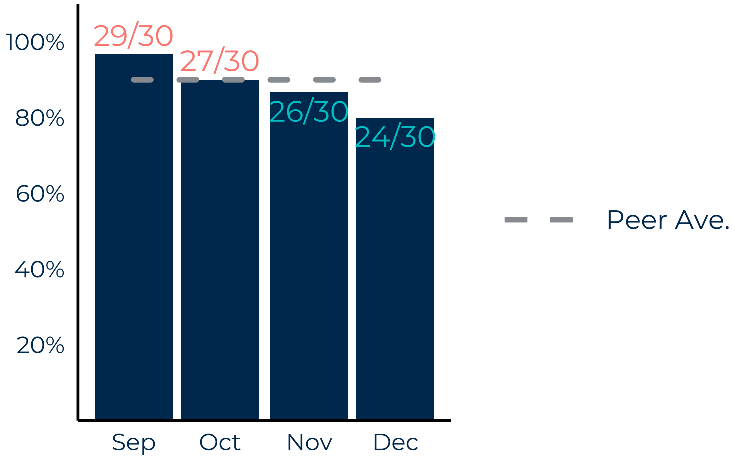

A case-by-case breakdown of your results are available at your clinical quality dashboard.

More information about the rationale for the measure PONV 03 and how it is calculated are available here.

Sincerely,

The MPOG Quality Team

…

Card 5B

Dear Alex,

You are no longer a top performer for the measure of postoperative nausea and vomiting (PONV 03):

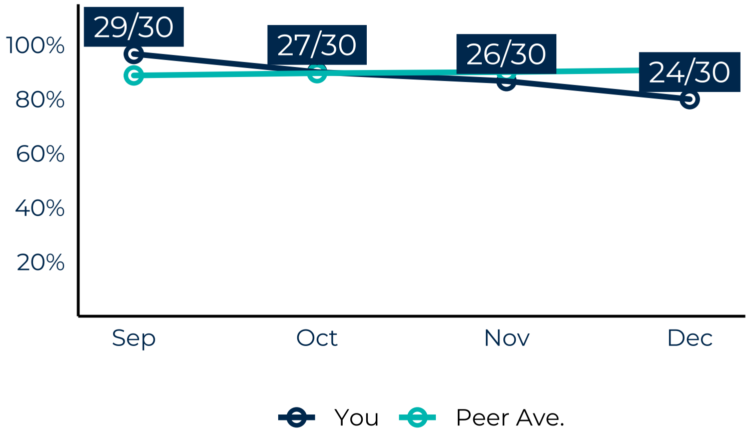

A case-by-case breakdown of your results are available at your clinical quality dashboard.

More information about the rationale for the measure PONV 03 and how it is calculated are available here.

Sincerely,

The MPOG Quality Team

Card 6A

Dear Alex,

Your performance dropped below the goal for the measure postoperative nausea and vomiting (PONV 03):

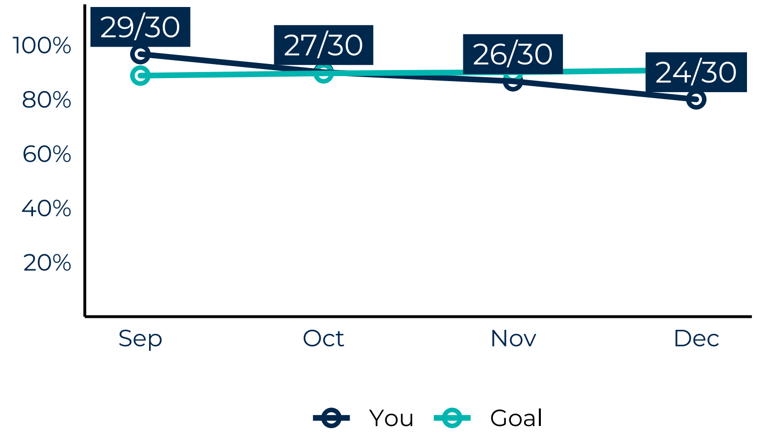

A case-by-case breakdown of your results are available at your clinical quality dashboard.

More information about the rationale for the measure PONV 03 and how it is calculated are available here.

Sincerely,

The MPOG Quality Team

…

Card 6B

Dear Alex,

Your performance dropped below the goal for the measure postoperative nausea and vomiting (PONV 03):

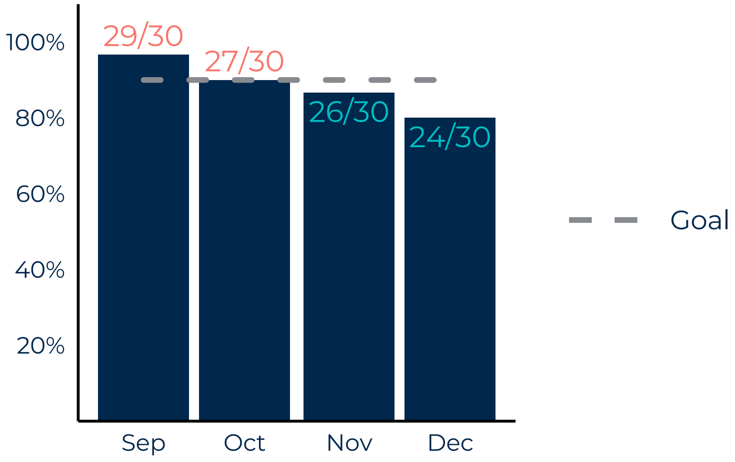

A case-by-case breakdown of your results are available at your clinical quality dashboard.

More information about the rationale for the measure PONV 03 and how it is calculated are available here.

Sincerely,

The MPOG Quality Team

Card 7A

Dear Alex,

You are not a top performer this month for the measure postoperative nausea and vomiting (PONV 03).

Your performance was 80% (24/30), below the peer average of 90% (27/30).

A case-by-case breakdown of your results are available at your clinical quality dashboard.

More information about the rationale for the measure PONV 03 and how it is calculated are available here.

Sincerely,

The MPOG Quality Team

…

Card 7B

Dear Alex,

You are not a top performer this month for the measure postoperative nausea and vomiting (PONV 03):

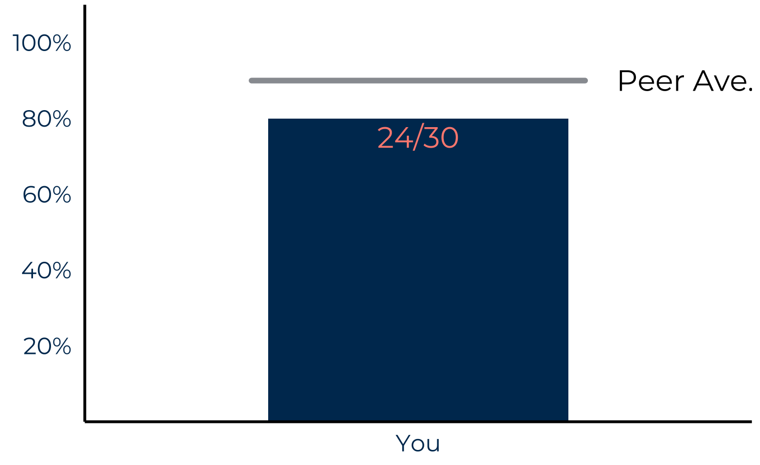

A case-by-case breakdown of your results are available at your clinical quality dashboard.

More information about the rationale for the measure PONV 03 and how it is calculated are available here.

Sincerely,

The MPOG Quality Team

Card 8A

Dear Alex,

You may have an opportunity to improve your performance for the measure postoperative nausea and vomiting (PONV 03):

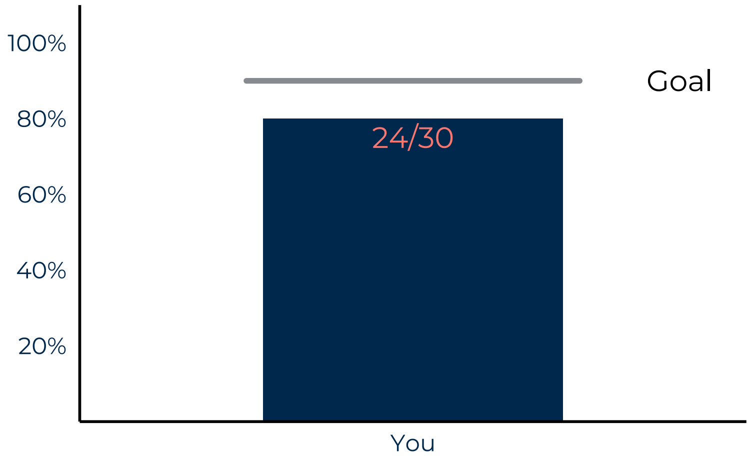

A case-by-case breakdown of your results are available at your clinical quality dashboard.

More information about the rationale for the measure PONV 03 and how it is calculated are available here.

Sincerely,

The MPOG Quality Team

…

Card 8B

Dear Alex,

You may have an opportunity to improve your performance for the measure postoperative nausea and vomiting (PONV 03).

This month your performance was 80% (24/30), below the goal of 90% (27/30).

A case-by-case breakdown of your results are available at your clinical quality dashboard.

More information about the rationale for the measure PONV 03 and how it is calculated are available here.

Sincerely,

The MPOG Quality Team

Card 9A

Dear Alex,

You are a consistent top performer for the measure of postoperative nausea and vomiting (PONV 03):

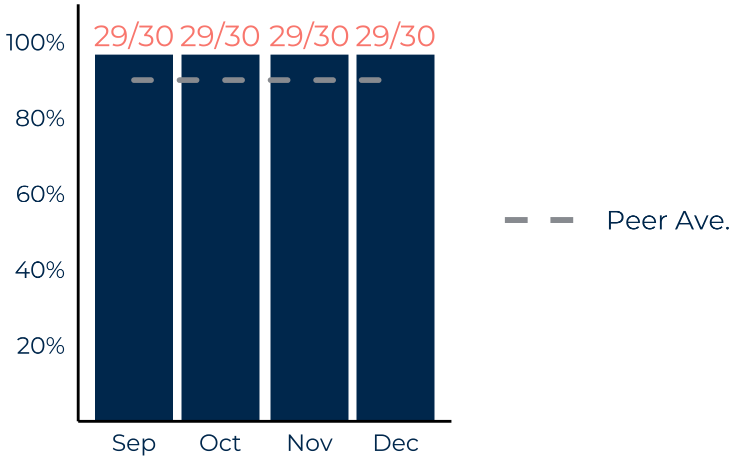

A case-by-case breakdown of your results are available at your clinical quality dashboard.

More information about the rationale for the measure PONV 03 and how it is calculated are available here.

Sincerely,

The MPOG Quality Team

…

Card 9B

Dear Alex,

You are a consistent top performer for the measure of postoperative nausea and vomiting (PONV 03):

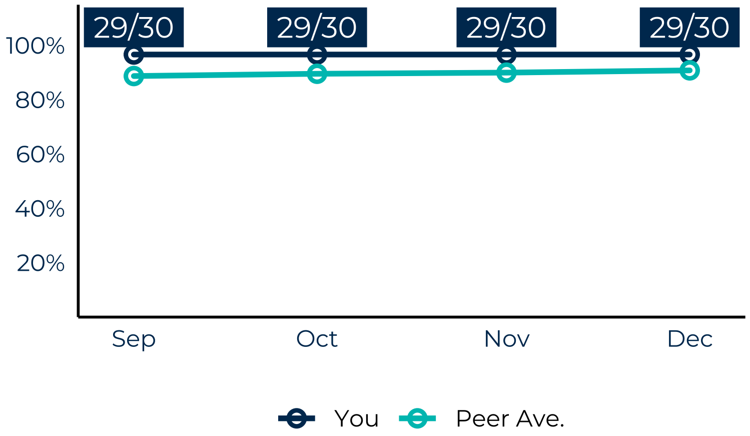

A case-by-case breakdown of your results are available at your clinical quality dashboard.

More information about the rationale for the measure PONV 03 and how it is calculated are available here.

Sincerely,

The MPOG Quality Team

Card 10A

Dear Alex,

Your performance is consistently high for the measure postoperative nausea and vomiting (PONV 03):

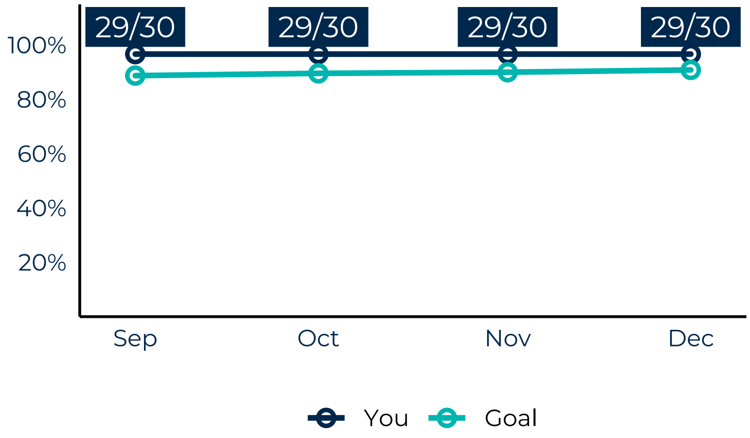

A case-by-case breakdown of your results are available at your clinical quality dashboard.

More information about the rationale for the measure PONV 03 and how it is calculated are available here.

Sincerely,

The MPOG Quality Team

…

Card 10B

Dear Alex,

Your performance is consistently high for the measure postoperative nausea and vomiting (PONV 03):

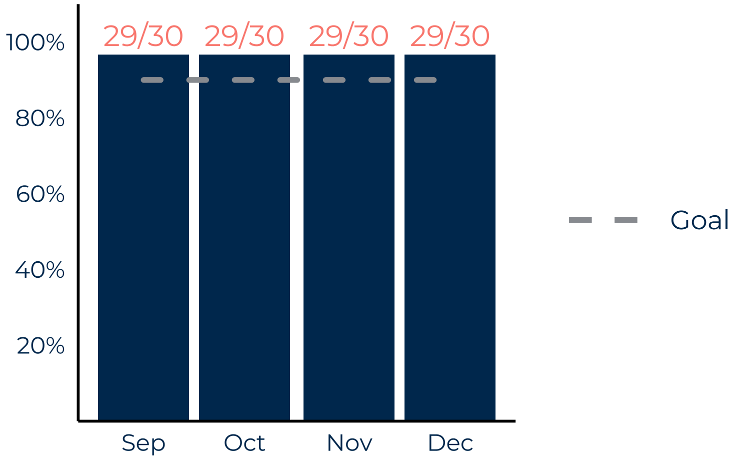

A case-by-case breakdown of your results are available at your clinical quality dashboard.

More information about the rationale for the measure PONV 03 and how it is calculated are available here.

Sincerely,

The MPOG Quality Team

Card 11A

Dear Alex,

You are a top performer this month for the measure postoperative nausea and vomiting (PONV 03).

Your performance was 96.7% (29/30), above the peer average of 90% (27/30).

A case-by-case breakdown of your results are available at your clinical quality dashboard.

More information about the rationale for the measure PONV 03 and how it is calculated are available here.

Sincerely,

The MPOG Quality Team

…

Card 11B

Dear Alex,

You are a top performer this month for the measure postoperative nausea and vomiting (PONV 03):

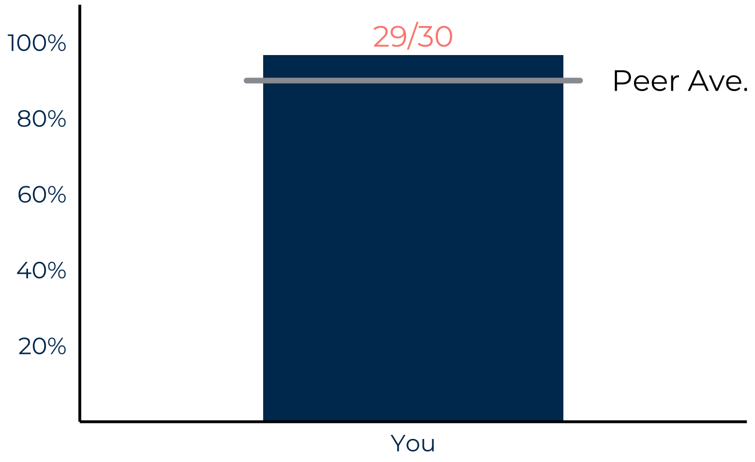

A case-by-case breakdown of your results are available at your clinical quality dashboard.

More information about the rationale for the measure PONV 03 and how it is calculated are available here.

Sincerely,

The MPOG Quality Team

Card 12A

Dear Alex,

Congratulations on your high performance for the measure postoperative nausea and vomiting (PONV 03):

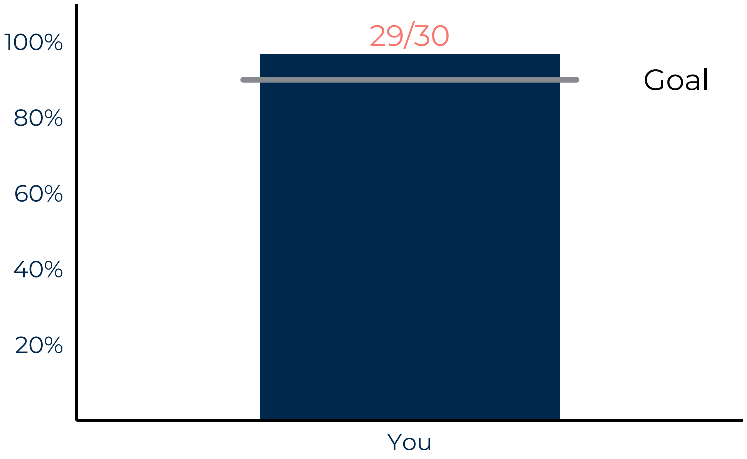

A case-by-case breakdown of your results are available at your clinical quality dashboard.

More information about the rationale for the measure PONV 03 and how it is calculated are available here.

Sincerely,

The MPOG Quality Team

…

Card 12B

Dear Alex,

Congratulations on your high performance for the measure postoperative nausea and vomiting (PONV 03).

Your performance was 96.7% (29/30), above the goal of 90% (27/30).

A case-by-case breakdown of your results are available at your clinical quality dashboard.

More information about the rationale for the measure PONV 03 and how it is calculated are available here.

Sincerely,

The MPOG Quality Team

Card 13A

Dear Alex,

Your performance has remained low for the measure postoperative nausea and vomiting (PONV 03):

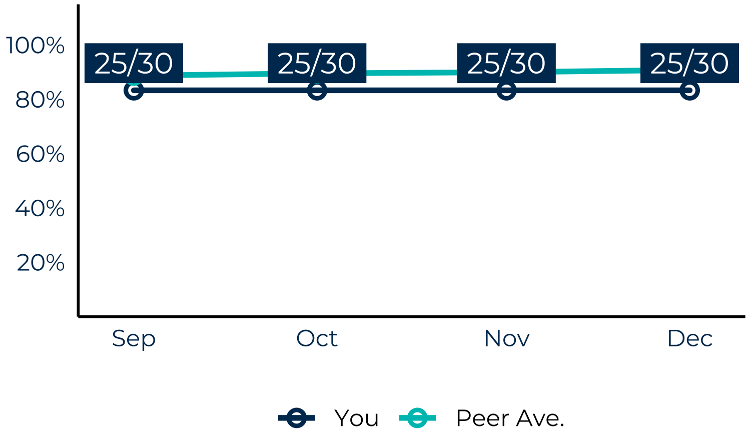

A case-by-case breakdown of your results are available at your clinical quality dashboard.

More information about the rationale for the measure PONV 03 and how it is calculated are available here.

Sincerely,

The MPOG Quality Team

…

Card 13B

Dear Alex,

Your performance has remained low for the measure postoperative nausea and vomiting (PONV 03):

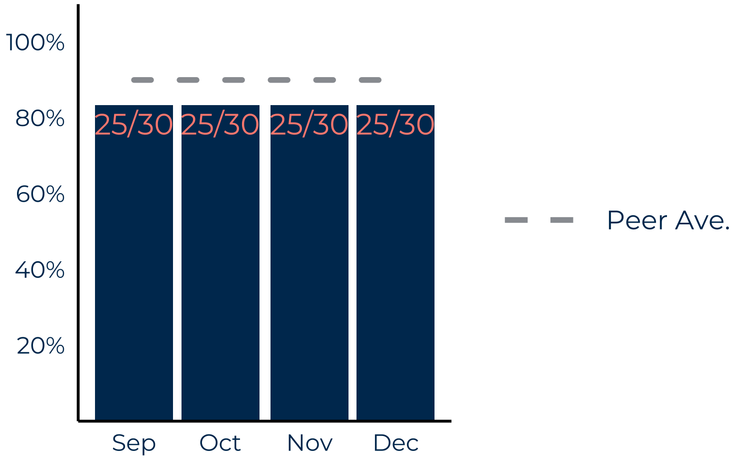

A case-by-case breakdown of your results are available at your clinical quality dashboard.

More information about the rationale for the measure PONV 03 and how it is calculated are available here.

Sincerely,

The MPOG Quality Team

Card 14A

Dear Alex,

Your performance has not improved for the measure of postoperative nausea and vomiting (PONV 03):

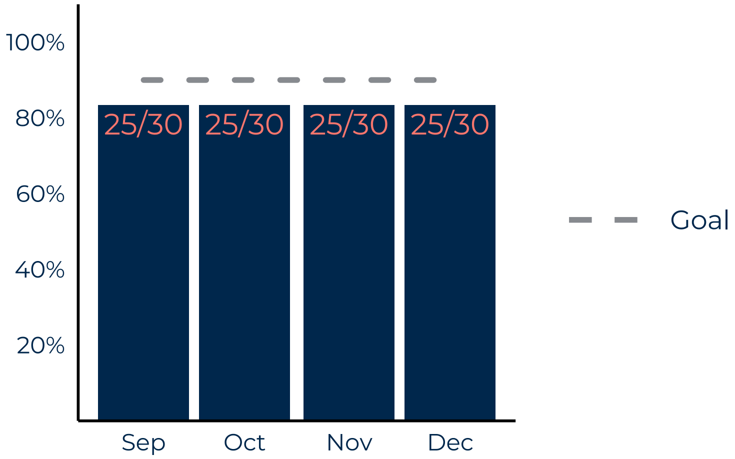

A case-by-case breakdown of your results are available at your clinical quality dashboard.

More information about the rationale for the measure PONV 03 and how it is calculated are available here.

Sincerely,

The MPOG Quality Team

…

Card 14B

Dear Alex,

Your performance has not improved for the measure of postoperative nausea and vomiting (PONV 03):

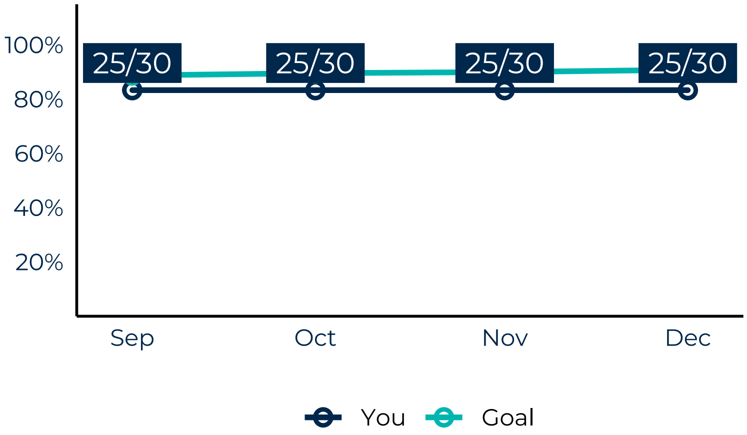

A case-by-case breakdown of your results are available at your clinical quality dashboard.

More information about the rationale for the measure PONV 03 and how it is calculated are available here.

Sincerely,

The MPOG Quality Team

Card 15A

Dear Alex,

You are not a top performer this month for the measure postoperative nausea and vomiting (PONV 03):

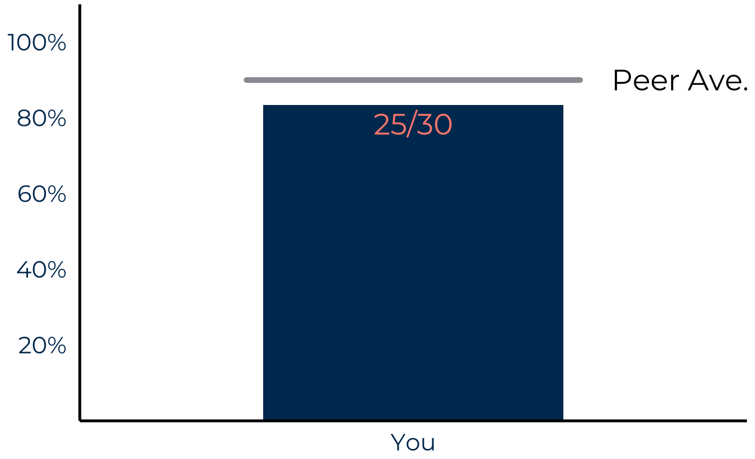

A case-by-case breakdown of your results are available at your clinical quality dashboard.

More information about the rationale for the measure PONV 03 and how it is calculated are available here.

Sincerely,

The MPOG Quality Team

…

Card 15B

Dear Alex,

You are not a top performer this month for the measure postoperative nausea and vomiting (PONV 03).

Your performance was 83.3% (25/30), below the peer average of 90% (27/30).

A case-by-case breakdown of your results are available at your clinical quality dashboard.

More information about the rationale for the measure PONV 03 and how it is calculated are available here.

Sincerely,

The MPOG Quality Team

Card 16A

Dear Alex,

You may have an opportunity to improve your performance for the measure postoperative nausea and vomiting (PONV 03).

This month your performance was 83.3% (25/30), below the goal of 90% (27/30).

A case-by-case breakdown of your results are available at your clinical quality dashboard.

More information about the rationale for the measure PONV 03 and how it is calculated are available here.

Sincerely,

The MPOG Quality Team

…

Card 16B

Dear Alex,

You may have an opportunity to improve your performance for the measure, postoperative nausea and vomiting (PONV 03):

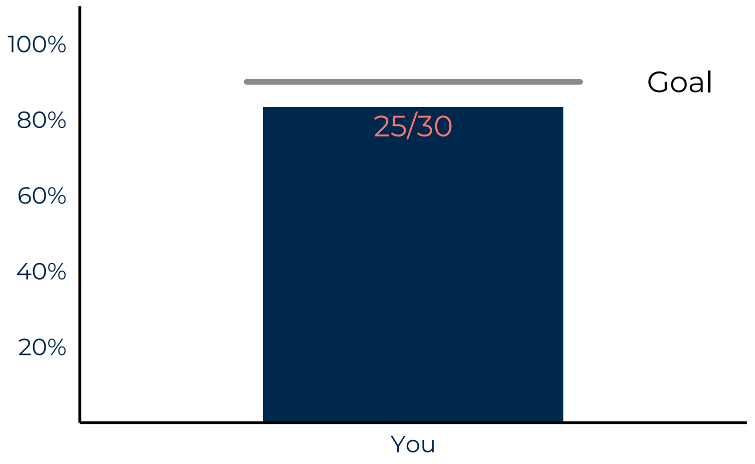

A case-by-case breakdown of your results are available at your clinical quality dashboard.

More information about the rationale for the measure PONV 03 and how it is calculated are available here.

Sincerely,

The MPOG Quality Team
